# AGITG MASTERPLAN: a randomised phase II study of modified FOLFIRINOX alone or in combination with stereotactic body radiotherapy for patients with high-risk and locally advanced pancreatic cancer

**DOI:** 10.1186/s12885-021-08666-y

**Published:** 2021-08-19

**Authors:** Andrew Oar, Mark Lee, Hien Le, Kate Wilson, Chris Aiken, Lorraine Chantrill, John Simes, Nam Nguyen, Andrew Barbour, Jaswinder Samra, Katrin M. Sjoquist, Alisha Moore, David Espinoza, Val Gebski, Sonia Yip, Julie Chu, Andrew Kneebone, David Goldstein

**Affiliations:** 1 Icon Cancer Centre, Gold Coast University Hospital, Gold Coast, Australia; 2 Liverpool and Macarthur Cancer Therapy Centres, Sydney, Australia; 3 Department of Radiation Oncology, Royal Adelaide Hospital, Adelaide, Australia; 4 NHMRC Clinical Trials Centre, University of Sydney, Sydney, Australia; 5 Wollongong Hospital, Wollongong, Australia; 6 Department of Gastroenterology and Hepatology, Royal Adelaide Hospital, Discipline of Medicine, University of Adelaide, Adelaide, Australia; 7 Princess Alexandra Hospital, Brisbane, Australia; 8 Royal North Shore Hospital, Sydney, Australia; 9 Cancer Care Centre, St George Hospital, Kogarah, Australia; 10 Trans-Tasman Radiation Oncology Group, Newcastle, Australia; 11 Peter MacCallum Cancer Centre, Melbourne, Australia; 12 Department of Radiation Oncology Royal North Shore Hospital Sydney Australia and University of Sydney, Sydney, Australia; 13 Department of Medical Oncology, Nelune Cancer Centre, Prince of Wales Hospital, Sydney, Australia

**Keywords:** Pancreatic cancer, Pancreas, Stereotactic radiotherapy, SBRT, Modified FOLFIRINOX, mFOLFIRINOX, Gemcitabine, *Nab*-paclitaxel, Borderline resectable

## Abstract

**Background:**

Among patients with non-metastatic pancreatic cancer, 80% have high-risk, borderline resectable or locally advanced cancer, with a 5-year overall survival of 12%. MASTERPLAN evaluates the safety and activity of stereotactic body radiotherapy (SBRT) in addition to chemotherapy in these patients.

**Methods and design:**

MASTERPLAN is a multi-centre randomised phase II trial of 120 patients with histologically confirmed potentially operable pancreatic cancer (POPC) or inoperable pancreatic cancer (IPC). POPC includes patients with borderline resectable or high-risk tumours; IPC is defined as locally advanced or medically inoperable pancreatic cancer. Randomisation is 2:1 to chemotherapy + SBRT (investigational arm) or chemotherapy alone (control arm) by minimisation and stratified by patient cohort (POPC v IPC), planned induction chemotherapy and institution. Chemotherapy can have been commenced ≤28 days prior to randomisation. Both arms receive 6 × 2 weekly cycles of modified FOLFIRINOX (oxaliplatin (85 mg/m^2^ IV), irinotecan (150 mg/m^2^), 5-fluorouracil (2400 mg/m^2^ CIV), leucovorin (50 mg IV bolus)) plus SBRT in the investigational arm. Gemcitabine+*nab*-paclitaxel is permitted for patients unsuitable for mFOLFIRINOX. SBRT is 40Gy in five fractions with planning quality assurance to occur in real time. Following initial chemotherapy ± SBRT, resectability will be evaluated. For resected patients, adjuvant chemotherapy is six cycles of mFOLFIRINOX. Where gemcitabine+*nab*-paclitaxel was used initially, the adjuvant treatment is 12 weeks of gemcitabine and capecitabine or mFOLFIRINOX. Unresectable or medically inoperable patients with stable/responding disease will continue with a further six cycles of mFOLFIRINOX or three cycles of gemcitabine+*nab*-paclitaxel, whatever was used initially. The primary endpoint is 12-month locoregional control. Secondary endpoints are safety, surgical morbidity and mortality, radiological response rates, progression-free survival, pathological response rates, surgical resection rates, R0 resection rate, quality of life, deterioration-free survival and overall survival. Tertiary/correlative objectives are radiological measures of nutrition and sarcopenia, and serial tissue, blood and microbiome samples to be assessed for associations between clinical endpoints and potential predictive/prognostic biomarkers. Interim analysis will review rates of locoregional recurrence, distant failure and death after 40 patients complete 12 months follow-up. Fifteen Australian and New Zealand sites will recruit over a 4-year period, with minimum follow-up period of 12 months.

**Discussion:**

MASTERPLAN evaluates SBRT in both resectable and unresectable patients with pancreatic ductal adenocarcinoma.

**Trial registration:**

Australia New Zealand Clinical Trials Registry ACTRN12619000409178, 13/03/2019.

Protocol version: 2.0, 19 May 2019

## Background

Pancreatic cancer (PC) is anticipated to become the second leading cause of cancer death in the United States by 2030 [1]. Surgery remains the only curative treatment option in patients with PC [2]. Unfortunately surgery is only feasible in between 15 and 20% of patients due to presence of metastatic disease, vascular involvement and/or significant comorbidities [3]. Furthermore, despite improvements in activity of adjuvant chemotherapy regimens, over 70% of resected patients will succumb [4]. The majority of patients without metastatic disease present with locoregionally advanced disease that can be classified as high-risk, borderline resectable (BRPC) or locally advanced pancreatic cancer (LAPC). For these patients the 5-year overall survival is only 12% [3]. Improved treatment paradigms in these patients with non-metastatic pancreatic cancer are desperately needed [5].

The current recommended management for patients with operable PC, including patients with high-risk features of size > 4 cm, extrapancreatic extension and node positivity, is resection with consideration of adjuvant chemotherapy [4]. The high metastatic rate seen in all patients with PC has seen recent interest in giving neoadjuvant chemotherapy in all high-risk patients [6–9]. Even with surgery, 40% of patients will experience a locoregional recurrence (LRR) in the first 12 months [10]. LRR is the most common site of treatment failure for patients with PC [4] and this is a major contributor to the substantial morbidity and mortality of this cancer [11]. The location of the pancreas in the upper abdomen means patients with PC often experience debilitating gastrointestinal symptoms including gastric outlet obstruction, biliary obstruction and/or pain. If attempts to ameliorate local progression of tumour do not increase cure or surgical resection rates, they still may prevent the significant morbidity that is associated with local tumour progression [12].

For BRPC and LAPC, the recommended treatment is for chemotherapy with or without external beam radiation therapy (EBRT), and consideration of surgical resection in those that become resectable [13]. Some institutions would recommend up front surgical resection followed by adjuvant chemotherapy +/− EBRT in those initially deemed to have BRPC [13, 14]. Recent results have indicated a significant improvement in R0 resection with neoadjuvant chemoradiotherapy and hypofractionated radiotherapy [15, 16]. Neoadjuvant FOLFIRINOX, with or without chemoradiotherapy has shown impressive R0 resection rates in small studies [17, 18]. However, there are significant toxicities associated with this regimen and several retrospective studies have shown that mFOLFIRINOX, with an initial dose reduction is associated with better safety profile and no change in efficacy [19]. ESPAC-5F randomised BRPC patients to neoadjuvant gemcitabine/capecitabine, FOLFIRINOX or conventional CRT with the final arm being immediate surgery. Patient numbers were too small to compare neoadjuvant approaches, however it did demonstrate an improvement in 1-year overall survival (OS) favouring neoadjuvant treatment over upfront surgery (77% vs 40%, *P* < 0.001) [20]. The SWOG S1505 results, presented at ASCO 2020, conducted a direct comparison between mFOLFIRINOX and gemcitabine/*nab*-paclitaxel as peri-operative treatment for pancreatic cancer. The authors concluded that there is little evidence that either treatment improved OS to historical standards [21]. This publication needs evaluation prior to making conclusions from this relatively small study. The optimal adjuvant treatment in patients undergoing resection remains controversial [22, 23]. Until recently, gemcitabine monotherapy was considered standard of care [24, 25]; however, the ESPAC-4 study demonstrated an OS benefit of adding capecitabine to gemcitabine monotherapy [4]. Adjuvant mFOLFIRINOX has shown superior outcomes to adjuvant gemcitabine monotherapy [26]. The importance of research into the peri-operative approach for the management of pancreatic cancer is supported in a recent editorial [27].

A systematic review on the use of SBRT in PC demonstrated encouraging results with a median OS of 17 months [28]. A large review in LAPC has suggested superior outcomes with SBRT, compared to chemotherapy and EBRT [11]. SBRT is a significant dose escalation to standard EBRT, and in non-small cell lung cancer randomised evidence has shown improved efficacy and reduced toxicity [29]. SBRT is anticipated to increase tumour cell kill and reduce rates of LRR [30]. SBRT prior to surgical resection has been demonstrated to be safe with no increase in surgical complications compared to EBRT [31]. Reduced LRR rates could increase the possibility of cure, reduce the debilitating symptoms associated with LRR and progression, and potentially improve OS by preventing or delaying development of metastases. The ultimate aim from a multimodality approach is to achieve an R0 resection and subsequently potential cure. A negative resection margin is a well-documented predictor of OS [32–34]. With SBRT, the shorter treatment course of 2 weeks compared to approximately 5 weeks with conventional EBRT, allows patients to proceed to surgery and further systemic treatment earlier.

It is essential that the apparent benefits of this technique be explored in a multi-centre randomised setting to provide solid evidence. The combination of chemotherapy and SBRT, while promising, remains unpublished in a randomised setting. At the time of this publication results from the A021501 Alliance Trial have just been presented, although not published, warranting comparison of trial methodologies [35, 36]. The chemotherapy regimens included in MASTERPLAN are included as accepted standard of care, during a time when standard of care is controversial and often institutional dependent.

## Methods/design

### Objectives

The MASTERPLAN study aims to evaluate the safety and activity of SBRT in addition to chemotherapy for the treatment of POPC and IPC. The primary objective of the MASTERPLAN randomised phase II study is to determine if the addition of SBRT to modern chemotherapy improves locoregional control for patients with POPC and IPC as defined by RECIST criteria v1.1 [37] and Australasian Gastrointestinal Trial Group (AGITG) guidelines for resectability [38]. Local and regional relapse will be determined based on imaging findings and/or biopsy (see *Assessment and Follow-Up*). Regional relapse is defined as disease progression to regional lymph nodes that would be included in a resection of pancreatic lesion in the same location (see *Surgery*). Patients will be randomized to chemotherapy +/− SBRT and be stratified by patient cohort (POPC versus IPC), planned chemotherapy regimen, chemotherapy commenced (Yes versus No [from up to 28 days prior to randomisation]), and institution. Staging of pancreatic cancer will be as per the AGITG guidelines for resectability, classifying patents as high-risk, borderline resectable or locally advanced. Potentially operable patients with high-risk or borderline resectable disease will be classified as POPC, and patients with locally advanced disease or medically inoperable pancreatic cancer will be classified as inoperable pancreatic cancer (IPC). Given the encouraging outcomes with mFOLFIRINOX chemotherapy, this will be the preferred regime (Option 1). Clinicians will have the option of administering gemcitabine and *nab*-paclitaxel (Option 2), and this will need to be specified prior to randomisation. Patients will be randomised in a 2:1 ratio to the investigational arm with six cycles of chemotherapy and SBRT or the control arm with six cycles of chemotherapy alone. Initial chemotherapy can have commenced ≤28 days prior to randomisation. SBRT will be 40Gy in five fractions administered over 2 weeks with central plan storage and pre-treatment quality assurance review through the Trans-Tasman Radiation Oncology Group (TROG).

Secondary outcomes include assessment of 1) Safety (frequency and severity of adverse events as defined by NCI CTCAE V4.03 and RTOG), 2) Surgical morbidity/mortality (length of stay, death within 30 days, frequency and severity of adverse events at 30 and 90 days post-surgery as defined by Clavien grading system), 3) Radiological response rates (RECIST v1.1), 4) Progression-free survival (PFS) (disease progression or death), 5) Pathologic response rates (tumour regression grade), 6) Surgical resection rates (rates of attempted resection excluding ‘open and close’ operations), 7) R0 resection rates (complete resection of gross tumour with negative surgical margins > 1 mm), 8) Quality of life (QoL) (EORTC QLQ C30 and PAN26 QoL), 9) Deterioration-Free Survival (DFS) (EORTC QLQ C30), and 10) OS (death from any cause).

Tertiary objectives for MASTERPLAN are to study associations between clinical endpoints and potential predictive/prognostic biomarkers (tissue, blood) including but not limited to ctDNA and protein signatures (association of biomarkers with clinical endpoints) and serial buccal and faecal microbial samples (variation in microbial composition with chemotherapy, correlation with response). Additionally, radiological assessments of nutrition and sarcopenia will be investigated in the RANDOMS sub-study.

### Eligibility

**Fig. 1:**
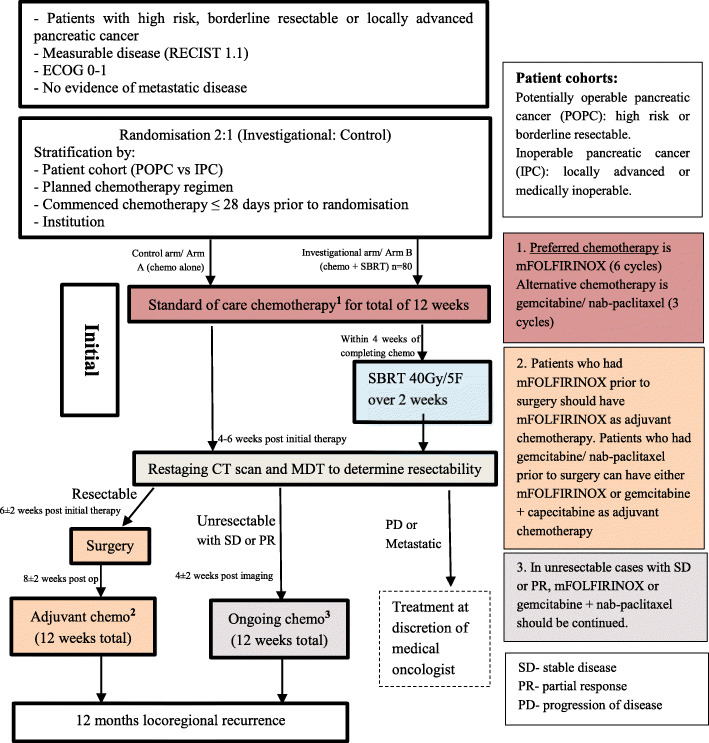
Masterplan study schema

After site credentialing, clinicians will be able to screen potential patients for eligibility during clinical review. Eligible patients must have one of the following: (1) T3 (tumour > 4 cm) (2) Extrapancreatic extension, (3) Node positive, (4) Borderline resectable pancreatic cancer *or* (5) LAPC. In addition, patients must: be aged 18–75 years, have histologic confirmation of pancreatic adenocarcinoma, have measurable disease according to RECIST v1.1, be of ECOG performance status 0 to 1, have study treatment planned to start within 14 days of registration, be willing and able to comply with all study requirements, and provide signed, written informed consent. Patients will need to have adequate renal, haematological and hepatic function defined as bilirubin less than 150% of the upper limit of normal (ULN) and AST/ALT less than five times the ULN. In patients who have had a recent biliary drainage and whose bilirubin is descending, a value of less than three times ULN is acceptable. Major exclusion criteria include: tumour size greater than 70 mm, duodenal invasion seen on endoscopy, prior abdominal radiotherapy, evidence of metastatic disease on baseline radiologic investigations, excluding non-melanomatous skin cancers, pre-invasive cervical cancer or early endometrial cancer, patients with a history of other malignancies are eligible if they have been continuously disease free for at least 2 years after definitive treatment, serological confirmed HIV positivity with CD4 count less than 400, concurrent illness, including severe infection that may jeopardise the ability of the patient to undergo the procedures outlined in this protocol with reasonable safety, neuroendocrine pancreatic carcinoma and life expectancy of less than 3 months. Baseline imaging including a computed tomography (CT) scan of chest/abdomen/pelvis with contrast and appropriate imaging of pancreas using a pancreatic protocol in addition to a ^18^F-FDG PET is required. Registration requires multidisciplinary confirmation of patient suitability by a medical oncologist, radiation oncologist, surgeon and radiologist. Central review of diagnostic (baseline) images will occur with site feedback of staging planned. Figure 1 below presents the study schema and patient flow.

### Trial governance

The study is sponsored by the AGITG and conducted by the National Health and Medical Research Council (NHMRC) Clinical Trials Centre (CTC), University of Sydney in collaboration with the TROG. This study will be performed in accordance with the Note for Guidance on Good Clinical Practice (Integrated Addendum to ICH E6 (R1): Guidelines for Good Clinical Practice ICH E6(R2) annotated with TGA comments (Therapeutic Goods Administration DSEB July 2000) and in compliance with applicable laws and regulations, NHMRC Statement on Ethical Conduct in Research Involving Humans 2007, NHMRC Australian Code for the Responsible Conduct of Research (updated 2015) and principles laid down by the World Medical Assembly in the Declaration of Helsinki 2008.

The study will be conducted in accordance with applicable Privacy Acts and Regulations. All data generated in this study will remain confidential. All information will be stored securely at the NHMRC CTC, University of Sydney. Radiotherapy data generated from the radiotherapy quality assurance activities will be stored securely at TROG. Prior to opening for recruitment, sites will undergo a credentialing process to obtain approval from TROG to confirm SBRT planning and treatment delivery capability. A separate assessment of radiotherapy plan quality and treatment compliance will be undertaken by TROG on study completion. No patient will be recruited to the study until all the necessary Human Research Ethics Committee and other approvals have been obtained and the patient has provided written informed consent.

### Initial treatment

#### Chemotherapy

Patients will receive either six cycles of mFOLFIRINOX (Option 1) or if unsuitable for mFOLFIRINOX, three cycles of gemcitabine and *nab*-paclitaxel (Option 2). mFOLFIRINOX will be six 14-day cycles of oxaliplatin (85 mg/m^2^ IV), irinotecan (150 mg/m^2^ IV), 5-fluorouracil (2400 mg/m^2^ CIV over 46 h), leucovorin (50 mg IV bolus) all given on Day 1. Alternatively, patients can have three cycles of gemcitabine (1000 mg/m^2^ IV) and *nab*-Paclitaxel (125 mg/m^2^ IV) Day 1, Day 8 and Day 15 per a 28-day cycle.

### Stereotactic body radiotherapy (SBRT)

Patients randomised to SBRT will receive repeat staging with CT chest/abdomen/pelvis with contrast after chemotherapy and prior to SBRT. Patients without progressive disease will receive SBRT as 40Gy in five fractions, 8Gy per fraction, with two to four fractions delivered per week over 2 weeks. Two consecutive days of SBRT are permitted but not 3 days. SBRT is to commence within 4 weeks of completing initial chemotherapy. Given this prescription dose is above threshold dose for nearby organs at risk (OAR), compromise of coverage will be required near these structures. A radiotherapy quality assurance (QA) planning manual has been developed and available to all sites. AGITG and TROG guidelines for contouring and simulation have been published for use in the MASTERPLAN study [39]. To aid with image guidance during SBRT, endoscopic ultrasound (EUS) guided fiducial insertion will be performed. At least 2–4 inert sterile gold bars (4 mm long × 0.5 mm thick) in a preloaded EUS fine aspiration needle (Cook Medical, USA) will be placed in or around the pancreatic mass to outline its border. An additional core biopsy will be taken at this time for further analysis as part of a translational sub-study.

During radiation planning and delivery, TROG will provide QA in accordance with TROG Policy Statement TP E6 (Quality Assurance Guidelines), a QA technical review will be undertaken for SBRT plans with remote technical audits conducted by an independent reviewer to ensure protocol compliance and appropriate SBRT delivery. The on-trial quality assurance for the trial will consist of Pre-Treatment Radiotherapy QA Technical Review and Post-Treatment Radiotherapy QA Technical Review.

Bloods will also be taken after initial chemotherapy +/− SBRT and prior to surgery to assess renal, haematological, and hepatic function.

### Evaluation for surgical resection

Prior to surgery, all patients require a clinical assessment, routine bloods, CA19.9 and CEA, toxicity assessment and QoL questionnaire completion. CT chest, abdomen and pelvis scan will be mandatory 4 weeks post-completion of chemotherapy +/− SBRT, with images transferred for central storage and batched independent, blinded assessment at a later stage.

### Surgery

For all patients deemed resectable, surgery should be performed 6 weeks (±2 weeks) after completion of the initial therapy (i.e. after completion of initial chemotherapy if patient randomised to control arm and after completion of SBRT in patients randomised to the investigational arm). The aim of the surgery is to achieve R0 resection. When the tumour is within the head of the pancreas, patients would be offered a standard Whipple’s procedure and level 2/3 dissection [40], with modification to obtain margin clearance. Assessment of preferred surgical method for Whipple’s procedure (open, laparoscopic, robotic) indicated nearly all surgeons expected to use the open approach in this study’s population. For lesions in the tail, patients will be offered standard modular resection as described by Strasberg and colleagues [41, 42]. For a distal pancreatectomy the majority of surgeons planned to use a laparoscopic approach unless an open approach is indicated, with robotic or open approaches noted as first preference for some surgeons. Segmental venous resection, as well as adjacent organ resection, can be performed at the time of standard, radical or extended radical pancreatoduodenectomy if required. The pylorus-preserving procedure is contraindicated only for carcinomas of the anterosuperior part of the head of the pancreas.

The standard pancreatoduodenectomy resection may comprise of regional lymphadenectomy around the duodenum and pancreas. For head/neck lesions this includes the lymph nodes on the right side of the hepatoduodenal ligament, the right side of the superior mesenteric artery (superior and inferior), and the anterior and posterior pancreaticoduodenal lymph nodes. For body/tail lesions this includes the pancreaticosplenic lymph nodes and lymph nodes to the left of superior mesenteric artery (superior and inferior). A lymphadenectomy beyond the abovementioned area could therefore be considered an extended lymphadenectomy. These anatomical definitions will be used to determine locoregional recurrence. If macroscopic removal of the tumour and/or clinically involved nodes is not possible, these patients will receive ongoing treatment as per trial protocol. If distant metastases are found at surgery, these patients should receive ongoing treatment at discretion of treating team. The method used for all surgical events will be prospectively obtained.

### Pathology

Standardised synoptic pancreas cancer histology reporting is required as outlined in Royal College of Pathologists of Australasia [43]. Post-surgery, the full histological details including the surgical staging; the presence and extent of residual viable tumour at the primary site and in the nodes will be recorded according to the AJCC 8th edition [44]. It is preferred that a minimum of 10 lymph nodes be examined and their status included in the final histopathological report. Primary tumour response to neoadjuvant therapy will be reported as the percentage of residual viable tumour cells compared with fibrotic stroma. Pathological complete response (pCR) rates will be recorded as per the College of American Pathology tumour regression grade (TRG). Tumour differentiation grade and the presence of lymphovascular invasion must also be reported. The histological assessment will determine the completeness of resection. A distance from tumour to all resection margins of 1 mm or greater will be regarded as complete resection (R0). All others will be considered an incomplete resection (R1). The measurement will be recorded in millimetres for verification by central histopathology review. Locoregional progression cannot be determined based on pathological assessment. For example, patients with radiologically node negative disease who are found to have node positivity on surgical resection specimens will not be considered to have locoregional progression. Patients in whom metastatic disease is identified pathologically (e.g., peritoneal metastasis) will be considered to have distant failure. Resection rates will exclude ‘open and close’ procedures. The rate of R0 resections will be compared between arms, and pre-treatment staging.

### Post-operative treatment for resectable patients

Following surgical resection, adjuvant chemotherapy should commence within 8 weeks (+/− 2 weeks) after completion of surgery. Patients having surgical resection should receive an additional six cycles (12 weeks) of adjuvant mFOLFIRINOX following surgery. In patients who received gemcitabine and *nab*-paclitaxel as initial chemotherapy, adjuvant chemotherapy may be mFOLFIRINOX or three cycles of gemcitabine and capecitabine (1000 mg/m^2^ gemcitabine administered intravenously on Days 1, 8 and 15 of a 4-week cycle with 1660 mg/m^2^ oral capecitabine in two divided doses orally on Days 1–21 per a 28-day cycle.

### Ongoing chemotherapy for patients not having resection

Patients determined not suitable for surgery after multidisciplinary review, either unresectable or medically inoperable, will continue with the initial choice of chemotherapy within 4 weeks (+/− 2 weeks) of imaging. This is a further six cycles of mFOLFIRINOX or where gemcitabine and *nab*-paclitaxel was used initially a further three cycles of gemcitabine and *nab*-paclitaxel.

Unresectable patients with locoregional progression or metastatic disease (progressive disease (PD)) at CT scan restaging may receive further chemotherapy at the discretion of their treating medical oncologist. Follow-up data until death will be collected.

### Assessment and follow-up

The primary endpoint of 12-month locoregional control will be assessed using RECIST version 1.1 criteria. A CT chest/abdomen/pelvis with contrast will be performed within 7 days prior to registration, prior to SBRT (SBRT arm only), 4–6 weeks following induction treatment (chemotherapy +/− SBRT), 4 weeks post-surgery (if applicable), then at 6, 9 and 12 months following study randomisation, and every 3 months during Year 2, every 6 months during Years 3 and 4, unless new disease is reported.

Patients who experience synchronous locoregional and distant progression will be deemed to have a locoregional failure. Local and regional relapse will be determined based on imaging findings and/or biopsy (if performed). The first post-treatment scan cannot be used to determine locoregional relapse due to treatment related changes that may impact imaging assessment. However, locoregional control can be determined and dated back to the first scan only if the subsequent scan confirms locoregional progression. Regional relapse is defined as disease progression to regional lymph nodes that would be included in a resection of pancreatic lesion in the same location (see *Surgery*). Given the fluctuating nature of regional lymphadenopathy, regional progression should be demonstrated on at least two sequential scans. If local or regional progression is demonstrated on a single scan and a change of therapy is initiated, or the patient deteriorates with no further imaging; local/regional progression should be considered at the date of the scan which resulted in treatment change or preceded the deterioration. In addition, a new tumour growth within the pancreas but separate to the primary lesion will be categorised as a regional failure. For large and equivocal recurrences, the epicentre of the recurrence will be the location to determine if local, regional or distant recurrence. All patients will be assessed for treatment related toxicity at all follow-up visits. The CTCAE v5.0 will be used to grade toxicity. Surgical morbidity and mortality will be assessed and using the Clavien grading system at discharge post-surgery, and at 30 days and 90 days. The length of hospital stay, calculated from day of surgery to date of discharge from acute care hospitalisation and will include intensive care admissions.

PFS will be calculated from the time of randomisation to the time of first documented clinical or imaging relapse or the date of death from any cause, whichever occurs first. Disease progression is defined according to RECIST v1.1.

Quality of life (QoL) QoL will be assessed using the European Organisation for the Research and Treatment of Cancer (EORTC) Quality of Life Questionnaires - QLQ C-30 and QLQ-PAN26.

Sarcopenia will be assessed using CT-assessed body composition analysis and completion by participant and local dietician of the Patient Generated – Subjective Global Assessment (PG-SGA) which will generate scores to be used in addition to objective radiological assessments of sarcopenia. These assessments will be performed by trained dietitians on centrally stored images as part of the RANDOMS sub-study (Radiological and subjective measures of nutrition, diet and sarcopenia).

### Schedule of assessments

Baseline assessment of patients includes clinical examination, haematological, serum electrolytes (creatinine / creatinine clearance) and liver function assessment along with tumour marker (CA19.9). Patient reported outcomes commence at this time with quality of life (EORTC QLQ C30 and PAN26 QoL) and dietary questionnaires (PG-SGA). An 18-FDG PET will be performed within 28 days of randomisation to exclude patients with metastatic disease.

Assessment during chemotherapy includes haematological, biochemistry and liver function assessments, repeated at Days 8 and 15 for patients receiving Option 2 chemotherapy (gemcitabine + *nab*-paclitaxel) and prior to each injection of gemcitabine for patients receiving gemcitabine + Capecitabine. CA19.9 is assessed at every treatment cycle, then every 3 months to 12 months post end of treatment. Toxicity and adverse events are assessed at each cycle.

A CT scan of chest, abdomen and pelvis will be performed within 14 days prior to randomisation then every 4–6 weeks post-completion of initial treatment (initial chemotherapy ± SBRT), prior to confirmation of eligibility for surgery and at 3, 6, 9 and 12 months from randomisation. Patients receiving SBRT will have CT 2 weeks prior to SBRT - 2 (±1 week) weeks following initial chemotherapy. CT images will be stored for central radiology review.

A CT scan must be performed prior to surgery and a CT chest, abdomen and pelvis scan is mandatory 4–6 weeks post-surgery. All surgical specimens are required for retrospective central histological assessment and reporting of resection outcome (R0 versus R1). Tumour tissue and normal tissue (pancreas and duodenum) for translational research is collected from all surgical patients at time of resection. Surgical complications are assessed per Clavien grading system at discharge, 30 and 90 days post-surgery.

Consultation with a radiation oncologist following chemotherapy is required to confirm the patient remains suitable for SBRT. For patients receiving SBRT, late radiation therapy AEs will be assessed every 3 months for the first year then 6-monthly up to 4 years.

Quality of life questionnaires are completed during chemotherapy, and prior to SBRT and 4–6 weeks post-completion of initial treatment (initial chemotherapy +/− SBRT). For patients eligible for surgery QoL is completed within 3 days prior to surgery. All patients will complete QoL questionnaires 30 days after end of treatment then at 3, 6, 9 and 12 months from randomisation and followed by completion every 6 months during Years 2, 3 and 4.

The Patient Generated – Subjective Global Assessment (PG-SGA) questionnaire is completed within questionnaire within 7 days of CT scans performed at baseline, 4–6 weeks post-completion of initial therapy, 12 weeks post-surgery and 6 months post-randomisation. Assessment by a dietician will occur at the same time.

Participants will be followed up for a minimum of 12 months and up to 4 years. After completion of study treatment, participants will be followed up at 6, 9 and 12 months from study randomisation then every 6 months during Years 2, 3 and 4.

### Translational research

Tissue and blood samples will be collected from all participants for translational research. Archival formalin-fixed paraffin-embedded diagnostic core biopsy will be collected for central histology review. Surgical resection tissue (tumour and normal for resectable patients) will be collected and processed into formalin-fixed paraffin-embedded tissue and some stored in preservative (RNAlater) where possible. In addition, core biopsy at time of fiducial insertion (for patients having SBRT) and disease progression will be collected from consenting patients (optional).

Serial blood collections (up to seven timepoints) for translational research will occur at baseline, prior to SBRT (Arm B only), insertion of fiducial markers (Arm B, optional), 4–6 weeks post-completion of initial treatment (initial chemotherapy ± SBRT), at surgery (optional, at selected sites), 6 and 12 months post-randomisation or at progression (whichever comes first). Bloods are processed to recover serum and plasma and frozen and some research bloods may be shipped to central laboratories in real time for analysis. Translational research studies may include the molecular and genetic drivers of PC, prognostic and predictive biomarkers for clinical endpoints including circulating tumour DNA and a funded study of glycoproteins (by mass spectrometry).

A microbiome sub-study of 40 to 60 patients aims to assess the effect of chemotherapy on microbial composition in patients with PC. Serial microbial samples will be obtained with patients collecting their own buccal and faecal samples before, during and after chemotherapy. The primary objective is to explore the variation in microbial composition with chemotherapy. Secondary objectives include correlating variation in microbial composition with response, exploring the impact of opportunistic use of antibiotic and healthy diet on the variation microbial composition and correlating buccal and faecal microbial composition.

### Power/statistics

Among the potentially operable patients with high-risk or BRPC (POPC), we expect a LRR rate at 12 months of 40% [10]. LRR is defined as locoregional recurrence/progression, with or /without surgical resection. With the addition of SBRT a 55% relative (22% absolute) reduction in the LRR rate would be of clinical interest. With 60 patients in a 2:1 randomised phase II study there will be 80% power with 95% confidence to rule out an uninteresting LRR rate of 40% in favour of 18% [45]. This sample size accounts for a 25% competing risk rate where the first event is a distant failure or death [46].

Among inoperable patients with LAPC or medically IPC, we expect a LRR rate at 12 months of 50% [4, 46] where LRR is defined as locoregional progression after initial therapy. With the addition of SBRT a 46% relative (23% absolute) reduction in the LRR would be of clinical interest. With 60 patients in a 2:1 randomised phase II study there will be 80% power with 95% confidence to rule out an uninteresting LRR rate of 50% in favour of 27% [45]. This sample size accounts for a 25% competing risk rate where the first event is distant failure or death [46].

A comparative analysis of chemotherapy and SBRT versus chemotherapy alone for the two groups will be performed. The combined analysis will assess 1) LRR rates and 2) PFS. One hundred twenty patients (80 versus 40): Using competing risk analysis the trial will have 80% power and 95% confidence (two-tailed comparison) to detect a HR of 0.469 on LRR. One hundred twenty patients will provide preliminary evidence on PFS. Assuming no difference between the treatment groups across groups A and B, a 95% CI will provide a lower bound for the similarity of the two treatments. The lower bound for a one-sided 95% CI for the HR of 1 is 0.71, based on 90% event rate in the chemotherapy alone group at 24 months [46].

After the first 40 patients have completed 12 months of follow-up an interim analysis for assessment of the rates of locoregional failure, distant failure (metastasis) and death will occur. In addition, the rate of distant failure/death prior to locoregional relapse will be monitored periodically in the pooled cohort and if required the sample size may be re-estimated.

### Independent data monitoring committee (IDMC)

An IDMC will meet at least 6-monthly to review patient safety, trial progress, the planned interim analysis results and the periodic assessment of rate of distant failure/death prior to locoregional relapse.

## Discussion

The role of radiotherapy, including SBRT, in PC is controversial. To date there is no published randomised evidence exploring the role of SBRT in pancreatic cancer. Institutional experience reports high local control rates and low toxicity, however confirmation in a randomised study remains elusive. Recent improvements in systemic treatment, have placed an increased importance on locoregional control in this difficult to manage disease. In this publication we describe an investigator initiated multi-centre phase II trial conducted by the AGITG in collaboration with TROG and the NHMRC CTC which was developed in collaboration by these groups through a series of clinical and academic meetings and workshops in Australia.

MASTERPLAN has emerged as an important trial exploring the utility of SBRT in patients with high-risk, borderline resectable or LAPC. In this patient cohort, locoregional recurrence risk is high and can be potentially mitigated by SBRT. Given the high radiation doses employed with SBRT, and the potential for significant toxicity, it is important this technique is explored in the setting of a prospective trial with appropriate QA and follow-up. Pancreatic SBRT in the context of a clinical trial with RT QA offers a tremendous opportunity to train radiation oncologist and provide constructive feedback to treating teams. A separate assessment of radiotherapy plan quality and treatment compliance will be undertaken by TROG on study completion.

This trial assesses whether a clinical improvement in locoregional control can be achieved with pancreas SBRT. Locoregional control remains an endpoint of value to both clinicians and patients. The impact of locoregional progression can be devastating with associated morbidity and/or death. The 2:1 randomisation allows rapid collection of prospective data within the interventional arm, while maintaining an appropriate control arm for comparison. The interim analysis after 40 patients allows early assessment of the primary endpoint, in addition to distant failure and death, and the IDSMC reviews will determine if closure of the study is required due to safety issues or treatment futility. Despite R0 resection rates being a readily accessible and important endpoint, this is not an appropriate endpoint for interrogation during the interim analysis.

Published guidelines for SBRT delivery, in addition to real time RT QA ensure that results from this trial will be robust, extrapolatable and generalisable.

Secondary endpoints allow for assessment of safety, surgical morbidity, pathological response, tumour control and quality of life, all comparable between treatment groups with or without SBRT. Importantly, a number of sub-studies in the MASTERPLAN trial including translational sciences in addition to radiologically assessed markers of sarcopenia, assessment of microbiome, provide a unique opportunity to collect a wealth of information and data across a range of disciplines.

A similar study Alliance for Clinical Oncology Trial A021501 [35, 36]has recently ceased the interventional arm after an interim analysis. That study randomised patients with BRPC to eight cycles of mFOLFIRINOX or seven cycles of mFOLFIRINOX + SBRT (interventional arm) and closed after interim analysis of 30 patients within each arm. The interim analysis revealed that the R0 resection rate was significantly less than 60% in the interventional arm, and therefore recruitment to that arm was ceased. Recruitment to the chemotherapy alone arm continues.

Results for Alliance A021501 were presented in early 2021 [36] and are yet to be fully published. It was reported neoadjuvant mFOLFIRINOX for patients with borderline resectable pancreatic cancer was associated with favourable OS relative to historical data. The study also found mFOLFIRINOX with hypofractionated RT did not improve OS compared to historical data. It was concluded that mFOLFIRINOX represents a reference regimen in this setting and a backbone on which to add novel agents.

There are some initial concerns regarding the heterogeneity between treatment groups in the A021501 publication. Patients in the interventional group had a baseline Ca19.9 that was nearly 50% higher (248 U/ml versus 171 U/ml). Fewer patients in the interventional arm underwent surgery (58% versus 51%), and an eventual pancreatectomy (48% versus 35%). These two factors have considerable impact on R0 resection status, the primary endpoint in determining the cessation of the interventional arm. Central review of resection margin status prior to cessation of the interventional arm in A021501 is controversial, due to the subjective nature of R0 resection status, the possibility of sterilised tumour cells impacting the R0 margin interpretation, and a low number of patients. Tumour cells considered viable near the resection margin may subsequently senesce and cloud reliability of margin status in patients receiving radiotherapy. Fewer patients going to surgery raises the possibility of discrepant radiological evaluation of patients following SBRT. It is well known that SBRT can make response assessment difficult. The 18-month OS in the interventional arm was 21% lower than the control arm (68% versus 48%) and when assessed amongst patients who underwent pancreatectomy it was 14% lower (93% versus 79%). However, it is noted the Alliance A021501 study was not powered to measure OS and this difference is most likely explained by fewer patients making surgery, a critical component of the treatment paradigm in patients with BRPC with significant impact on survival.

Pancreas SBRT is technically challenging even in high volume centres, with risks and consequences of geographical miss and/or exceeding normal tissue constraints. Therefore, MASTERPLAN mandates radiotherapy plan undergoing pre-treatment expert review, permitting corrections to suboptimal radiotherapy voluming and planning prior to treatment delivery. Contouring guidelines including an anatomic atlas, in addition to a planning and delivery manual were developed to further improve the standard of SBRT delivery. Patient eligibility for MASTERPLAN is much broader than that for A021501. MASTERPLAN includes patients with more earlier staging in the ‘high-risk’ cohort, and also those with less favourable anatomic features and with LAPC. Therefore, the applicability of MASTERPLAN patients to the general clinic may be greater.

MASTERPLAN addresses an important clinical question in a very challenging and increasingly common disease.

### Summary

This prospective randomised phase II study addresses the efficacy of SBRT in addition to modern chemotherapy in pancreatic cancer patients. Results from this study will inform the need for a larger phase III study. Importantly, MASTERPLAN provides an exciting platform for tissue, blood and microbiome collection for significant translational research into this devastating disease.

## Data Availability

Data sharing is not applicable to this article as no datasets were generated or analysed during the current study.
